# Identifying relapse predictors in individual participant data with decision trees

**DOI:** 10.1186/s12888-023-05214-9

**Published:** 2023-11-13

**Authors:** Lucas Böttcher, Josefien J. F. Breedvelt, Fiona C. Warren, Zindel Segal, Willem Kuyken, Claudi L. H. Bockting

**Affiliations:** 1 Frankfurt School of Finance and Management, Frankfurt am Main, Germany; 2 Department of Medicine, University of Florida, Gainesville, FL USA; 3 Department of Psychiatry, Amsterdam University Medical Center, University of Amsterdam, Amsterdam, the Netherlands; 4 NatCen Social Research, London, UK; 5 Institute of Health Research, College of Medicine and Health, University of Exeter, Exeter, UK; 6 Department of Clinical Psychological Science, University of Toronto Scarborough, Toronto, Ontario Canada; 7 Department of Psychiatry, University of Oxford, Oxford, UK

**Keywords:** Depression, Relapse, Individual participant data, Meta analysis, Machine learning, Decision tree, Logistic regression, Gradient boosting

## Abstract

**Background:**

Depression is a highly common and recurrent condition. Predicting who is at most risk of relapse or recurrence can inform clinical practice. Applying machine-learning methods to Individual Participant Data (IPD) can be promising to improve the accuracy of risk predictions.

**Methods:**

Individual data of four Randomized Controlled Trials (RCTs) evaluating antidepressant treatment compared to psychological interventions with tapering ($N=714$) were used to identify predictors of relapse and/or recurrence. Ten baseline predictors were assessed. Decision trees with and without gradient boosting were applied. To study the robustness of decision-tree classifications, we also performed a complementary logistic regression analysis.

**Results:**

The combination of age, age of onset of depression, and depression severity significantly enhances the prediction of relapse risk when compared to classifiers solely based on depression severity. The studied decision trees can (i) identify relapse patients at intake with an accuracy, specificity, and sensitivity of about 55% (without gradient boosting) and 58% (with gradient boosting), and (ii) slightly outperform classifiers that are based on logistic regression.

**Conclusions:**

Decision tree classifiers based on multiple–rather than single–risk indicators may be useful for developing treatment stratification strategies. These classification models have the potential to contribute to the development of methods aimed at effectively prioritizing treatment for those individuals who require it the most. Our results also underline the existing gaps in understanding how to accurately predict depressive relapse.

**Supplementary Information:**

The online version contains supplementary material available at 10.1186/s12888-023-05214-9.

## Background

Depression is one of the most prevalent mental conditions worldwide [1], and the COVID-19 pandemic may have further accelerated its rise [2]. Many individuals who suffer from depression experience a relapse of depressive episodes, even in spite of interventions such as continuation of antidepressants. It would be valuable to be able to identify individuals with a high risk of relapse, so that these individuals can be offered more intensive interventions or more careful monitoring. A recent Individual Participant Data Meta-Analysis (IPDMA) [3, 4] of randomized trials of antidepressant therapy versus psychological interventions while tapering antidepressants found that a younger age at onset, shorter duration of remission, and higher levels of depressive symptoms were associated with a higher overall risk of relapse. Importantly, this study did not find any moderators (i.e., factors that would indicate that one treatment type is more preferable for some patients compared to others).

In clinical psychiatry, “depressive relapse” is defined as the re-emergence of a depressive episode before remission during which the patient fulfills the criteria of a depressive disorder. The term “depressive recurrence” is typically used to describe the onset of a new depressive episode in patients who have already recovered [5–7]. For a more detailed discussion on relapse and recurrence, see, e.g., [8] and references therein. In this study, we will use the term “relapse” to describe a significant worsening of depressive symptoms both prior to and following a patient’s recovery.

Risk factors for depression relapse include severity of depressive symptomatology [3, 6, 7, 9–11], age of onset of depression [3, 6, 9, 10], number of previous depressive episodes, time in remission [3], anxiety disorders [12–15], dysfunctional attitudes [16], neuroticism [7, 16], cortisol levels [17], childhood maltreatment [7], and comorbid psychiatric disorders [3].

Depression scales such as Beck Depression Inventory (BDI) [18] and Hamilton Depression Rating (HAMD) [19] can be employed to estimate the risk of relapse in patients upon intake. Although depression scales may provide a possibility to predict relapse status, it would be desirable to use all factors that are available before the initiation of treatment and improve classification performance. For example, a recent work [20] has shown that certain multivariable prediction models had a better discrimination performance than a simple HAMD-based classifier. Here, we re-analyze an IPD sample of four Randomized Control Trials (RCTs) from [3] using decision trees to identify who is at high risk of relapse when starting relapse prevention treatment based on different individual characteristics. To study the robustness of the classification results obtained with different decision trees, we also perform a complementary logistic regression analysis.

Decision trees are a class of machine learning algorithms and have found application in computational psychiatry for the identification of decision pathways and their predictive value [21–28]. If applied to relapse prevention, decision trees can take into account predictors and their inter-dependencies to identify a specific subgroup of individuals (e.g., young females, with high residual symptoms) that have an elevated relapse risk at intake.

While decision trees have already found various applications in medicine, including diagnosis of type 2 diabetes [29], dengue disease [30], and cancer [31], their application in computational psychiatry to inform treatment selection has been limited.

Still, there is promise that decision trees are useful for improving clinical decision making in psychiatry [27, 28]. For example, decision trees have shown higher sensitivity and specificity compared to logistic regression in predicting major depressive disorder [25, 26]. In addition, decision trees found applications in predicting suicide risk [24], quality of life [21], late life depression [22, 23], and the effect of neuroticism and self-esteem on depression disorders [32]. One advantage of decision trees over other classification methods is that they are easily interpretable and closely resemble decision protocols that are common in medical diagnosis [33].

## Methods

The IPD [3, 34–37] that we analyze in this study comprises data of $N=714$ participants [mean (SD) age: 49.2 [11.5] years; 522 (73.1%) female] from 4 RCTs that compared the effectiveness of antidepressant monotherapy and two alternative psychological treatments, preventive Cognitive Behavioral Therapy (CBT) and Mindfulness-based Cognitive Therapy (MBCT), during and/or after antidepressant tapering. We included 10 risk indicators: Age (years), age of onset of depression (years), past episodes (number), HAMD (total score), BDI (total score), marital status (divorced/single/married), time since last episode (months), education level (degree/subdegree/no qualifications), psychiatric comorbidities (yes/no), and number of sessions. For all study participants, a censored follow-up period of 14 months was implemented. The binary outcome variable (i.e., the relapse status of a patient) was determined using a blinded clinical diagnostic interview [38, 39]. For all studies, it was required that participants are in remission and on antidepressant medication before randomization. In two studies, remission was determined based on the criterion that patients must have a maximum HAMD score of 7 [35] or 10 [37]. Patients were considered to be in remission for either an unspecified duration [36] or a minimum of 6 [6, 34] to 8 [35] months. Similar to previous work [3], our emphasis has been on complete patient data at follow-up, encompassing cases where all patient records were accessible and patients either experienced relapse or did not.

**Table 1:** Baseline demographic and clinical patient characteristics. The educational level “subdegree” indicates that qualifications are below degree level. We use the acronyms MADM (Maintenance Antidepressant Medication), PCT (Preventive Cognitive Therapy), and ADM+ (Tapering and/or Stopping Antidepressant Medication). This table is adapted from [3]

Characteristic	No. Participants	Prop. Participants	Mean (SD)	Total No. (range)
Female sex	522	73.1%	n/a	714 (n/a)
Male sex	192	26.9%	n/a	714 (n/a)
Presence of a comorbid psychiatric condition	252	38.2%	n/a	660 (n/a)
Marital status				
Partner	437	61.2%	n/a	714 (n/a)
Single	139	19.5%	n/a	714 (n/a)
Divorced, separated, or widowed	138	19.3%	n/a	714 (n/a)
Intervention type				
MADM	369	51.7%	n/a	714 (n/a)
MBCT while ADM+	287	40.2%	n/a	714 (n/a)
PCT while ADM+	58	8.1%	n/a	714 (n/a)
Educational level				
Degree	252	35.6%	n/a	707 (n/a)
Subdegree	378	53.5%	n/a	707 (n/a)
No qualifications	77	10.9%	n/a	707 (n/a)
Age	714	n/a	49.2 (11.5) years	714 (19–79)
Mean age at onset	708	n/a	26.9 (12.8) years	708 (4–70)
Previous episodes	624	n/a	5.6 (3.4)	624 (2–40)
Time in remission since last episode	660	n/a	17.5 (23.5) months	660 (0–178)
HAMD score at baseline	714	n/a	4.5 (4.1)	714 (0–20)

An overview of baseline demographic and clinical patient characteristics is provided in Table 1. After removing incomplete baseline observations from the dataset, we are left with 543 participants who possess complete baseline data. We use this subset of 543 participants to train decision-tree and logistic classifiers. In alignment with the complete dataset, the subset maintains a balanced distribution of both relapse and non-relapse patients.

When applicable, we followed the TRIPOD recommendations for developing and validating the models presented in this study [40]. The binary decision trees that we train and analyze are based on the Classification and Regression Trees (CART) model [41] as implemented in the Python library scikit-learn. We use the Gini criterion to identify features and thresholds that are associated with the largest information gain at each node in the decision tree. To test the performance of the employed classifiers, we train and test them on 1000 cross-validation realizations that consist of 70% (380 samples) and 30% (163 samples) of the given data, respectively. Since the number of participants with and without relapse is almost balanced (369 vs. 345) in the IPD that we use in this work, there is no need to implement correction methods for imbalanced datasets [42]. In addition to studying multi-feature CART models, we employ a reference classifier that solely relies on HAMD scores.

For a performance comparison, we use a logistic regression model and a gradient-boosting algorithm [43], which combines multiple decision trees to improve performance. Prior to training the logistic regression model, we standardize all input features to allow for a clearer interpretation and comparison of regression coefficients associated with different factors.

Before focusing on the decision-tree analysis, we study the effect of treatment type on relapse risk by comparing the observed proportions of relapse patients to a simple null model that assumes that there is an equal chance of experiencing relapse in both treatment classes. If the null model cannot be rejected (i.e., if treatment class is not associated with significant variations of relapse risk in the overall study population) with high confidence, we can exclude “treatment type” as a predictor of relapse risk during training.

## Results

There was no significant difference in the probability of relapse between the antidepressant and psychological treatment groups ($p = 0.12$). Among the 369 patients in the antidepressant group, 198 (53.7%) experienced relapse, while 171 (49.6%) relapsed in the psychological treatment group. As a result, our primary focus will be on relapse classification in a dataset without treatment stratification. In the Supplemental Information (SI), we provide results on classifier performance and feature importance for data that are stratified by treatment class. We show that decision trees achieve better classification results in the traditional treatment class compared to the alternative treatment class (Supplemental Fig. S1). Furthermore, our analysis in the SI reveals that HAMD is a more important feature for relapse prediction than BDI in the psychological treatment class, while the opposite is true in the antidepressant treatment class (Supplemental Fig. S2).

**Fig. 1:**
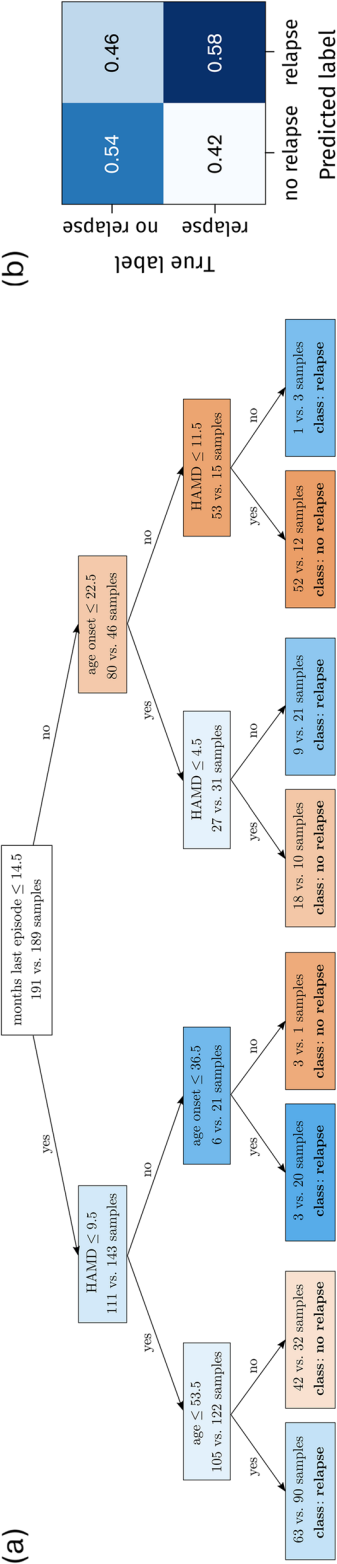
Decision tree–based multi-factor analysis. **a** A decision tree with a depth of three was trained on a dataset of 380 patient samples. In each node, the notation “*X* vs. *Y* samples” represents the counts of *X* non-relapse and *Y* relapse patients. The nodes are color-coded as orange or blue, denoting the dominant group in terms of non-relapse or relapse patients. The leaf nodes display the labels “relapse” or “no relapse”, indicating the predictions associated with the corresponding decision paths. The values of “age” and “age of onset” are provided in years. **b** Normalized confusion matrix associated with the decision tree shown in panel (**a**)

**Fig. 2:**
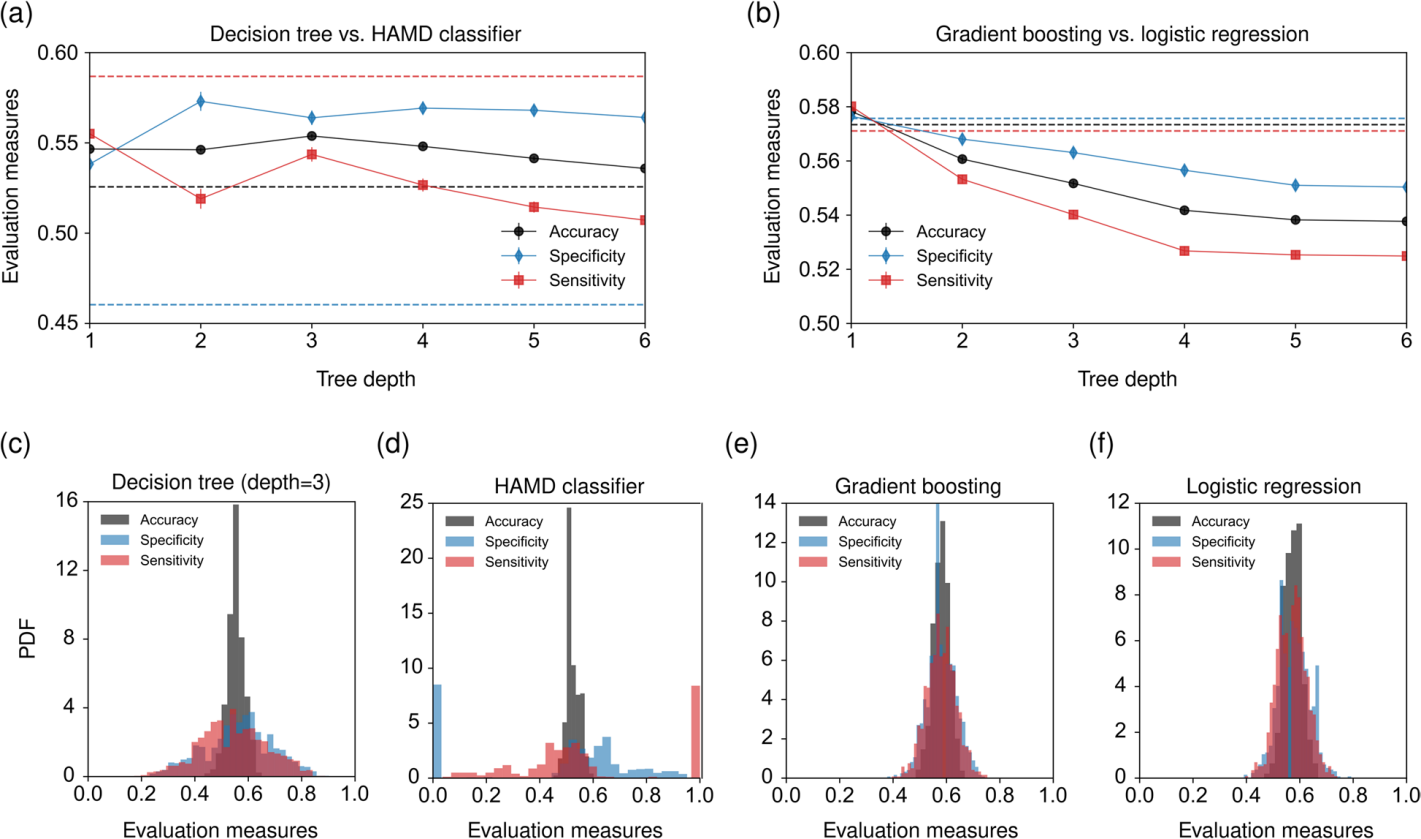
Performance comparison. **a**, **b** Accuracy (black disks), specificity (blue diamonds), and sensitivity (red squares) as a function of tree depth [in (**a**) for basic decision trees and in (**b**) for gradient-boosted trees]. Dashed lines in panels (**a**) and (**b**) represent the corresponding performance indicators of a classifier that is based on the HAMD score at intake and logistic regression, respectively. The training and test datasets consist of 380 and 163 samples, respectively. Markers in panels (**a**, **b**) indicate mean values that have been obtained using 1000 cross-validation realizations. Error bars indicate the corresponding standard errors. **c**–**f** Distributions of accuracy, specificity, and sensitivity for different classifiers

Figure 1 shows a decision tree with a depth of three and its corresponding confusion matrix.^1^ Each node specifies one decision criterion associated with a factor like age or number of previous depressive episodes. Nodes are colored either blue or orange, depending on whether they classify patients as ones with relapse or no relapse, respectively. The decision tree in Fig. 1(a) classifies relapse status using age, age of onset of depression, HAMD, and the number of months since the last depressive episode. For the given test data, 58% of relapse patients are correctly classified as experiencing relapse of depression after treatment, and 54% of non-relapse patients are correctly classified as experiencing no relapse of depression [Fig. 1(b)]. In other words, the sensitivity and specificity of the shown classifier are 58% and 54%, respectively.

The decision tree shown in Fig. 1(a) represents a single instance selected from a collection of 1000 cross-validated trees. We conduct a cross-validation analysis to evaluate the performance of decision-tree classifiers with varying depths. The corresponding training and test datasets comprise 380 and 163 samples, respectively. We vary the tree depth from one to six and calculate

1 $$\begin{aligned} \textrm{accuracy}=\frac{\textrm{TP}+\textrm{TN}}{\textrm{TP}+\textrm{TN}+\textrm{FP}+\textrm{FN}}\,, \end{aligned}$$

2 $$\begin{aligned} \textrm{sensitivity}=\frac{\textrm{TP}}{\textrm{TP}+\textrm{FN}}\,, \end{aligned}$$

and

3 $$\begin{aligned} \textrm{specificity}=\frac{\textrm{TN}}{\textrm{TN}+\textrm{FP}}\,, \end{aligned}$$

for each instance. Here, the quantities $\textrm{TP}$, $\textrm{TN}$, $\textrm{FP}$, and $\textrm{FN}$ denote true positives (i.e., “relapse” identified as “relapse”), true negatives (i.e., “no relapse” identified as “no relapse”), false positives (i.e., “no relapse” identified as “relapse”), and false negatives (i.e., “relapse” identified as “no relapse”), respectively. In addition to monitoring accuracy, sensitivity, and specificity, studying performance measures such as positive predictive value (PPV) and negative predictive value (NPV) can provide more insights into a classifier’s effectiveness, especially when considering the prevalence of a condition. For a balanced dataset, which we consider in our study, PPV and NPV can be directly calculated from sensitivity and specificity values (see, e.g., [44]).

As shown in Fig. 2(a), a tree of depth of three is associated with a good balance between high accuracy, specificity, and sensitivity scores. The decision-tree generalization performance deteriorates for larger depths because of overfitting. For comparison with a classification that is solely based on a depression-scale evaluation, we trained a second decision tree that only uses HAMD scores [dashed lines in Fig. 2(a)]. Although the sensitivity of such a classifier is larger than that of a multi-factor decision tree with a depth of three (0.587 vs. 0.543), we find that both accuracy and specificity are substantially smaller (0.526 and 0.460 vs. 0.554 and 0.564). The distribution of accuracy, specificity, and sensitivity of decision trees with a depth of three is unimodal and centered around values of about 0.55 [Fig. 2(c)]. However, a large proportion of the HAMD classifiers that we evaluated on 1000 cross-validation realizations label all patients as relapse patients and thus achieve a high sensitivity at the expense of specificity [Fig. 2(d)].

Since no HAMD score values were missing in the original dataset, we conducted the aforementioned HAMD-based classification on all 714 participants. Additionally, we assessed the performance of this classifier on the dataset with complete baseline data, consisting of 543 participants, which was used for training the decision tree models. The accuracy of the HAMD classifier on this dataset is 0.520, similar to the accuracy observed on the larger dataset. The specificity and sensitivity values are 0.843 and 0.194, respectively.

The performance of decision trees can be improved by combining multiple trees via gradient-boosting algorithms [43]. Figure 2(b) shows the performance of gradient-boosted trees for different tree depths. We observe that the accuracy, specificity, and sensitivity reach their maximum values when the tree depth is set to one. As a baseline for comparison, we train a logistic classifier and find that its performance measures are slightly smaller ($\textrm{accuracy}=0.573$, $\textrm{specificity}=0.576$, and $\textrm{sensitivity}=0.571$) than those of the most effective boosted tree ($\textrm{accuracy}=0.578$, $\textrm{specificity}=0.577$, and $\textrm{sensitivity}=0.580$).

We show the distributions of all three evaluation measures for gradient-boosted trees and logistic regression in Fig. 2(e,f). Both methods generate unimodal distributions that have narrower widths compared to those associated with a basic decision-tree classifier with a depth of three [Fig. 2(c)]. Although the overall performance of logistic classifiers and gradient-boosted trees is better than that of a basic decision tree, the latter may be more useful in certain clinical settings where human decision makers are relying on transparent and easily interpretable decision tools.

To evaluate the sensitivity of the decision-tree models in handling missing data, we utilized a *k*-nearest neighbor imputer with $k=2$ and uniform weights [45] to fill in the missing baseline values within the dataset. We then performed a decision tree analysis on the imputed dataset. Consistent with our earlier findings on decision trees without gradient boosting, we observed a favorable balance of accuracy (0.544), specificity (0.519), and sensitivity (0.567) for a tree depth of three. Similarly, for a gradient-boosted tree, we again found that a tree depth of one provided a satisfactory balance of accuracy (0.571), specificity (0.498), and sensitivity (0.639) scores.

**Table 2:** Overview of mean logistic-regression coefficients and mean odds ratios associated with standardized features. The values in parentheses denote 95% confidence intervals (CIs)

Quantity	Coefficient	Odds ratio
HAMD (intake)	0.254 (0.112, 0.400)	1.293 (1.119, 1.492)
Past episodes	0.214 (0.088, 0.361)	1.241 (1.091, 1.434)
Psychological comorbidities	0.097 $(-0.044,0.246)$	1.105 (0.957, 1.279)
Marital status	0.065 $(-0.047,0.189)$	1.069 (0.954, 1.208)
Education level	0.039 $(-0.083,0.158)$	1.042 (0.920, 1.171)
BDI (intake)	0.032 $(-0.110,0.180)$	1.035 (0.896, 1.197)
Number of sessions	-0.031 $(-0.153,0.092)$	0.971 (0.858, 1.096)
Age	-0.157 $(-0.279,-0.028)$	0.856 (0.756, 0.972)
Age of onset of depression	-0.204 $(-0.345,-0.074)$	0.817 (0.708, 0.929)
Months since last depressive episode	-0.226 $(-0.408,-0.096)$	0.800 (0.665, 0.908)

For logistic regression, the mean regression coefficients and mean odds ratios associated with all standardized input features are summarized in Table 2. We find that the most dominant factors in terms of an elevated relapse risk are HAMD, number of past episodes, and psychological comorbidities. The relapse risk decreases with the number of months since the last depressive episode, age of onset of depression, and age. Interestingly, the regression coefficient associated with the standardized BDI is almost eight times smaller than that of the standardized HAMD. In the SI, we discuss some of the underlying reasons for this observation. Our analysis of the BDI and HAMD distributions, conditioned on relapse status, shows that HAMD exhibits a higher level of discrimination regarding relapse status compared to BDI (Supplemental Fig. S3).

**Fig. 3:**
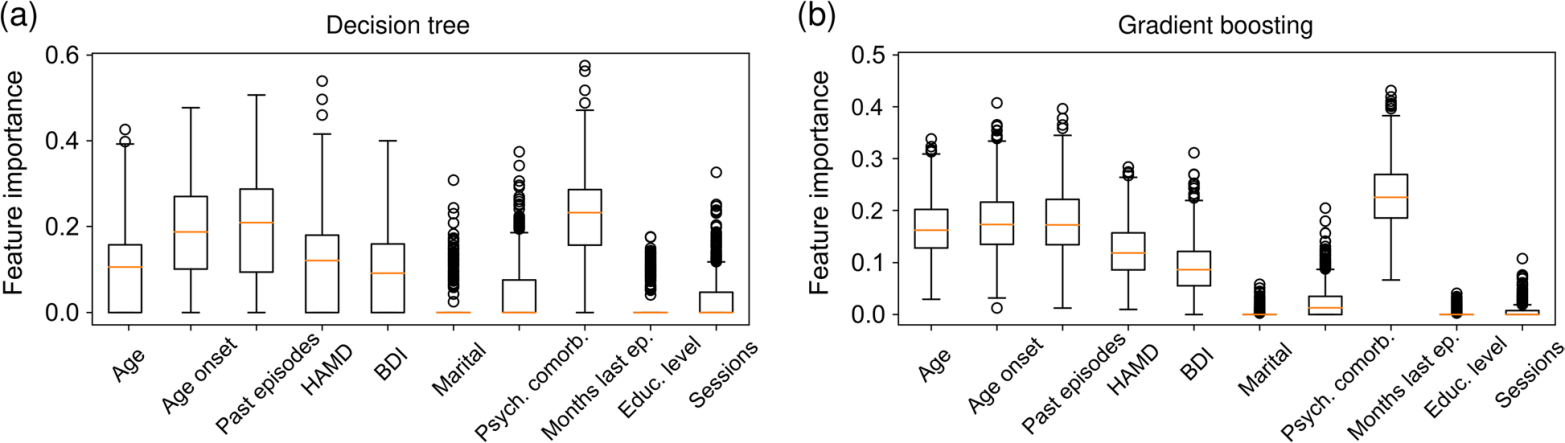
Decision tree feature importance. **a** Feature importance (i.e., the relative frequency at which a certain feature occurs in a trained decision-tree classifier) associated with a decision tree with a depth of three. **b** Feature importance associated with a gradient-boosted tree of depth one. The shown results are based on 1000 cross-validation realizations. The training dataset consist of 380 samples. In both box plots, red lines show the median feature importance. Outliers are represented by black circles

Figure 3 shows the relative frequency at which factors occur in trained decision trees (i.e., feature importance). In accordance with the logistic regression analysis, the most important factors are age, age of onset of depression, HAMD score at intake, number of past depressive episodes, and months since the last depressive episode.

## Discussion

We performed a multi-factor analysis of IPD ($N=714$) using decision trees to classify relapse status based on different demographic and clinical characteristics. We observed favorable performance in decision trees with a depth of three, achieving accuracy, specificity, and sensitivity scores approximately in the range of 54–56%. Further improvements were observed by employing gradient-boosting techniques, which enhanced these performance measures to values around 58%. Additionally, logistic regression yielded comparable levels of accuracy, specificity, and sensitivity.

In general, we found age, age of onset of depression, and months since the last depressive episode to be useful predictors of relapse. Also HAMD scores were identified by both decision trees and logistic regression as relevant relapse predictors. These results are in accordance with previous studies that also found age of onset of depression [3, 6, 9, 10], time in remission [3], and severity of the underlying depressive disorder [3, 6, 7, 9–11] to be relevant factors for identifying relapse patients. Psychological comorbidities were not identified as important features in the decision tree and logistic regression models. However, it is worth noting that another study [3] reported comorbid psychiatric disorders as influential factors in determining the time to relapse.

While based on relatively small sample sizes, the treatment-stratified analysis in the SI provides further insights into factors that are relevant to identify relapse patients. The analysis indicates that the number of past episodes and BDI scores are important features for predicting relapse in the traditional treatment class, but not in the alternative treatment class. Interestingly, BDI scores appear more frequently in the trained relapse classifiers for this class, whereas HAMD scores are more relevant predictors in the alternative treatment class. Furthermore, the treatment-stratified results suggest that decision trees can achieve higher accuracy, specificity, and sensitivity in the traditional treatment class compared to the alternative treatment class. Similar observations have been made in a recent study [20] that used elastic-net regression models to predict relapse.

Finally, we would like to discuss potential limitations that should be considered when interpreting and applying our findings that are not based on a pre-registered protocol. While our current analysis utilized datasets of a limited size, conducting further investigations using larger datasets (e.g., routine patient data) would provide valuable opportunities for studying potential applications of decision trees in computational psychiatry. Additionally, in our analysis of different classification methods, we utilized cross-validation with a 70/30 train-test split ratio. Exploring alternative split ratios and different decompositions of training and test data could prove valuable. For instance, it would be worthwhile to investigate training the model on a specific number of trials while evaluating its performance on the remaining studies. Moreover, we primarily focused on training our classification models on a subset of patients with complete baseline data. Hence, it would be beneficial to explore and compare different imputation methods designed to handle missing data.

Regarding the application of decision trees to identifying recurrent depression, it is worth noting that this study serves as a “proof-of-concept” and demonstrates that decision trees can provide visual insights into depression prediction, potentially benefiting clinicians in the future. However, it is important to approach the interpretation of the results with care, considering the potential for further improving model performance.

Furthermore, our results highlight the existing gaps in understanding how to accurately predict depressive relapse, which has been acknowledged by other researchers as well [9, 46].

## Conclusions

Classifying patients according to their relapse risk before the initiation of prevention treatment can be useful to improve clinical practice. While standard depression scales such as HAMD and BDI provide starting points to estimate relapse risk, our work shows that the overall predictive performance of relapse risk classifiers can be improved if multiple factors are combined. Decision trees are a class of algorithms capable of extracting important features and generating easily interpretable decision criteria from high-dimensional datasets. Our results indicate that decision trees can improve upon HAMD-based relapse prediction in terms of better accuracy and specificity. Gradient boosting techniques can further improve prediction performance by combining multiple trees into an ensemble. Boosted trees and logistic regression classifiers that used the same factors had comparable levels of accuracy, specificity, and sensitivity.

In summary, decision trees offer easily interpretable decision criteria and hold potential in aiding the development of methods that can identify individuals at high risk of relapse at intake, considering various individual characteristics. To enhance the robustness of classification results and further analyze such methods, training and testing these classifiers on larger datasets (e.g., routine patient data) would be desirable. In the context of clinical decision support, selecting a well-performing model from a cross-validation analysis can serve as a starting point. The subsequent steps involve adding more trial data and evaluating the performance of decision-tree classifiers using larger datasets, such as patient records. With the availability of more data, clinicians can continually refine and enhance the model.

### Supplementary Information

**Additional file 1.**

## Data Availability

The datasets generated and/or analysed during the current study are not publicly available due to patient consent restrictions but are available from the corresponding author on reasonable request.

## Abbreviations

ADM+: Tapering and/or Stopping Antidepressant Medication
BDI: Beck Depression Inventory
CART: Classification and Regression Trees
CBT: Cognitive Behavioral Therapy
CI: Confidence Interval
FN: False Negatives
FP: False Positives
HAMD: Hamilton Depression Rating
IPD: Individual Participant Data
IPDMA: Individual Participant Data Meta-Analysis
MADM: Maintenance Antidepressant Medication
MBCT: Mindfulness-based Cognitive Therapy
PCT: Preventive Cognitive Therapy
RCT: Randomized Control Trial
SD: Standard Deviation
TN: True Negatives
TP: True Positives

## Footnotes

1 A confusion matrix is a summary table that evaluates the performance of a given classification model by comparing its predictions to the actual outcomes. It shows true positives, true negatives, false positives, and false negatives, providing insights into a classifier’s effectiveness.
