# Supplementary material for: Identifying relapse predictors in individual participant data with decision trees
Source: BMC Psychiatry. 2023 Nov 13;23:835. doi: 10.1186/s12888-023-05214-9 (PMC10644580; doi:10.1186/s12888-023-05214-9)
Supplement: Supplementary file 1 — Additional file 1. [file 12888_2023_5214_MOESM1_ESM.pdf]

SUPPLEMENTAL INFORMATION
Identifying Relapse Predictors in Individual Participant Data with Decision Trees

Lucas Böttcher*

Department of Computational Science and Philosophy, Frankfurt School of Finance and Management, Frankfurt am Main, Germany† and Department of Medicine, University of Florida, Gainesville, FL, USA

Josefien J. F. Breedvelt*

Department of Psychiatry, Amsterdam University Medical Center, University of Amsterdam, Amsterdam, the Netherlands and NatCen Social Research, London, United Kingdom

Fiona C. Warren

Institute of Health Research, College of Medicine and Health, University of Exeter, Exeter, United Kingdom

Zindel Segal

Department of Clinical Psychological Science, University of Toronto Scarborough, Toronto, Ontario, Canada

Willem Kuyken

Department of Psychiatry, University of Oxford, Oxford, United Kingdom

Claudi L. H. Bockting

Department of Psychiatry, Amsterdam University Medical Center, University of Amsterdam, Amsterdam, the Netherlands

(Dated: August 9, 2023)

I. TREATMENT STRATIFICATION

Here, we study the impact of treatment stratification on decision-tree performance. Figure S1 shows the decision-tree performance as a function of tree depth for traditional and alternative treatment. After data cleaning (*i.e.*, removing incomplete baseline observations), the training and test datasets consist of 166 and 72 samples (traditional treatment) and 213 and 92 samples (alternative treatment). As in the main text, we observe that a good balance between high accuracy, specificity, and sensitivity scores is achieved for a tree with a depth of three. For the traditional treatment class, slightly larger performance values can be achieved because the underlying proportion of relapse patients is 53.7% while it is 49.6% in the alternative treatment class.

Consistent with the findings on feature importance discussed in the main text, our analysis reveals that age, age of onset of depression, and months since the last episode are utilized by

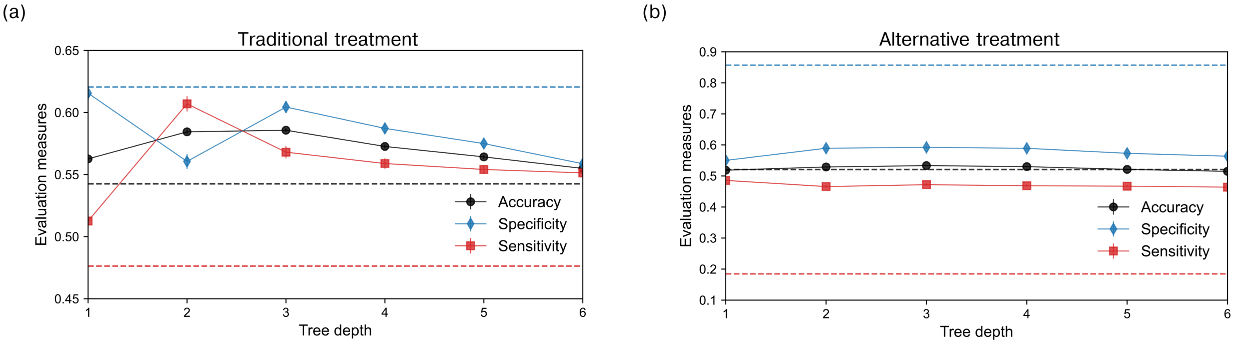

FIG. S1. Decision-tree performance under treatment stratification. Accuracy (black disks), specificity (blue diamonds), and sensitivity (red squares) as a function of tree depth [in (a) for traditional treatment and in (b) for alternative treatment]. Dashed lines represent the corresponding performance indicators of a classifier that is based on the HAMD score at intake. The training and test datasets consist of 166 and 72 samples (traditional treatment) and 213 and 92 samples (alternative treatment). Markers indicate mean values that have been obtained using 1000 cross-validation realizations. Error bars indicate the corresponding standard errors.

* These authors contributed equally.

† l.boettcher@fs.de

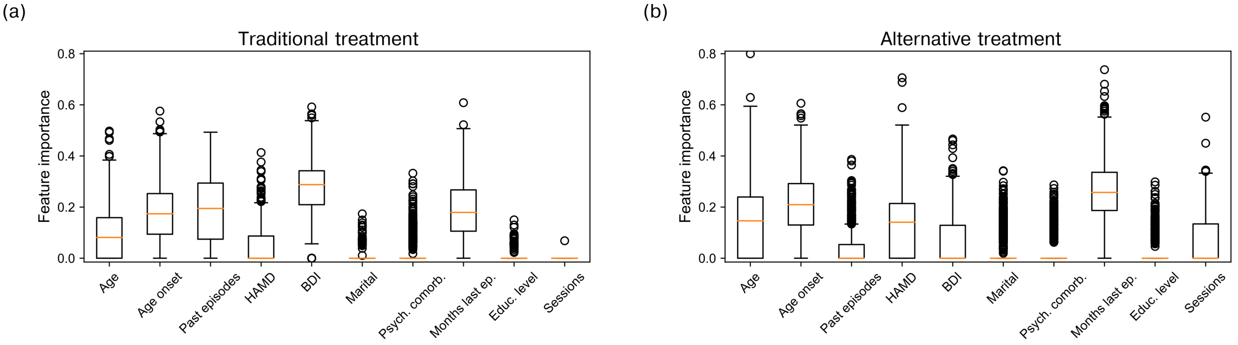

FIG. S2. Decision tree feature importance under treatment stratification. (a) Feature importance (*i.e.*, the relative frequency at which a certain feature occurs in a trained decision-tree classifier) associated with a decision tree with a depth of three and traditional treatment data. (b) Feature importance associated with a decision tree of depth three and alternative treatment data. The shown results are based on 1000 cross-validation realizations. The training dataset consist of 380 samples. In both box plots, red lines show the median feature importance. Outliers are represented by black circles.

a decision-tree classifier with a depth of three to predict the relapse status in both treatment groups (Figure S2). In the traditional treatment class, BDI is a more frequently selected predictor of relapse status (*i.e.*, a factor with higher feature importance) than HAMD while the opposite holds for the alternative treatment class. The number of past depressive episodes is a relevant predictor in the traditional treatment class. Because of the small sample sizes in the stratified data, many feature importance data points in Figure S2 appear as statistical outliers. Larger datasets would thus be required to provide more robust classification results.

II. BDI AND HAMD SCORES

We now compare the ability of BDI and HAMD surveys to identify patients with a high risk of relapse before the initiation of therapy. In particular, we examine if BDI and HAMD scores are useful measures to identify patients with an elevated relapse risk.

To study the ability of BDI and HAMD surveys to identify high-risk relapse patients at intake,

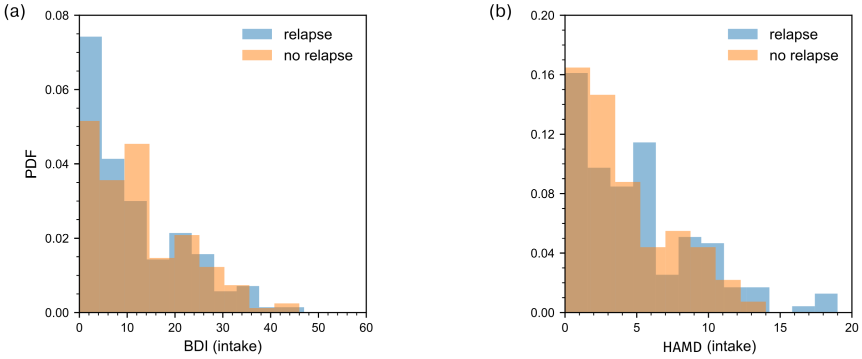

FIG. S3. Distributions of BDI and HAMD scores conditioned on relapse status.

we analyze corresponding distributions that are conditioned on relapse status (Figure S3). A difference in BDI and HAMD scores between relapse patients and non-relapse patients should be visible in the underlying, conditioned distributions. We therefore study the differences between BDI and HAMD distributions in terms of two non-parametric two-sample statistics: (i) the two-sample Kolmogorov–Smirnov (KS) test [1] and (ii) the Wilcoxon rank-sum test [2, 3]. Both methods test the hypothesis that two sets of samples are drawn from the same distribution. The null hypothesis is that the two distributions are identical.

The mean BDI scores at intake are 13.9 (relapse) and 11.6 (no relapse). The corresponding mean HAMD scores are 5.1 (relapse) and 3.9 (no relapse). These differences in mean values indicate that both survey types are able to identify differences between relapse patients and non-relapse patients before the initiation of treatment. To further test this hypothesis, we compute the corresponding KS and rank-sum statistics (Table I). For the BDI distributions, the p-values are 0.055 (KS test) and 0.065 (rank-sum test). For the HAMD distributions, they are 0.024 (KS test) and 0.004 (rank-sum test). The null hypothesis, stating that the distributions of associated with relapse and non-relapse patients are equivalent, can be rejected with a higher level of confidence for HAMD ($p < 0.05$) compared to BDI ($p > 0.05$), suggesting that HAMD scores are more indicative of relapse status at intake. This result is aligned with the findings provided in the main text.

**BDI**	KS test	0.113 (0.055)
	Rank-sum test	1.844 (0.065)
**HAMD**	KS test	0.126 (0.024)
	Rank-sum test	2.911 (0.004)

TABLE I. Comparing distributions of BDI and HAMD scores associated with relapse and non-relapse patients at intake with respect to the KS-test and the Wilcoxon rank-sum test. p-values are reported in parentheses.

[1] J. L. Hodges, The significance probability of the Smirnov two-sample test, Arkiv för Matematik **3**, 469 (1958).

[2] H. B. Mann and D. R. Whitney, On a test of whether one of two random variables is stochastically larger than the other, The Annals of Mathematical Statistics , 50 (1947).

[3] F. Wilcoxon, Individual comparisons by ranking methods, in *Breakthroughs in Statistics* (Springer, 1992) pp. 196–202.
